# Tissue- and column-specific measurements from multi-parameter mapping of the human cervical spinal cord at 3 T

**DOI:** 10.1002/nbm.3022

**Published:** 2013-09-16

**Authors:** RS Samson, O Ciccarelli, C Kachramanoglou, L Brightman, A Lutti, DL Thomas, N Weiskopf, CAM Wheeler-Kingshott

**Affiliations:** a NMR Research Unit, Queen Square MS Centre, Department of Neuroinflammation, UCL Institute of NeurologyQueen Square, London, UK; b NMR Research Unit, Queen Square MS Centre, Department of Brain Repair and Rehabilitation, UCL Institute of NeurologyQueen Square, London, UK; c University of CambridgeCambridge, UK; d Wellcome Trust Centre for Neuroimaging, UCL Institute of NeurologyQueen Square, London, UK; e Neuroradiological Academic Unit, Department of Brain Repair and Rehabilitation, UCL Institute of NeurologyQueen Square, London, UK

**Keywords:** spinal cord, magnetisation transfer, multi-parameter mapping, relaxometry

## Abstract

The aim of this study was to quantify a range of MR parameters [apparent proton density, longitudinal relaxation time *T*_1_, magnetisation transfer (MT) ratio, MT saturation (which represents the additional percentage MT saturation of the longitudinal magnetisation caused by a single MT pulse) and apparent transverse relaxation rate *R*_2_*] in the white matter columns and grey matter of the healthy cervical spinal cord. The cervical cords of 13 healthy volunteers were scanned at 3 T using a protocol optimised for multi-parameter mapping. Intra-subject co-registration was performed using linear registration, and tissue- and column-specific parameter values were calculated. Cervical cord parameter values measured from levels C1–C5 in 13 subjects are: apparent proton density, 4822 ± 718 a.u.; MT ratio, 40.4 ± 1.53 p.u.; MT saturation, 1.40 ± 0.12 p.u.; *T*_1_ = 1848 ± 143 ms; *R*_2_* = 22.6 ± 1.53 s^–1^. Inter-subject coefficients of variation were low in both the cervical cord and tissue- and column-specific measurements, illustrating the potential of this method for the investigation of changes in these parameters caused by pathology. In summary, an optimised cervical cord multi-parameter mapping protocol was developed, enabling tissue- and column-specific measurements to be made. This technique has the potential to provide insight into the pathological processes occurring in the cervical cord affected by neurological disorders. © 2013 The Authors. *NMR in Biomedicine* published by John Wiley & Sons, Ltd.

## INTRODUCTION

The spinal cord (SC) is a common site of involvement in neurological disorders, such as multiple sclerosis 1–3, amyotrophic lateral sclerosis 4, spinal cord injury (SCI) 5 and neuromyelitis optica 6. High-field post-mortem MRI studies have demonstrated focal and diffuse abnormalities in cord white matter (WM) and grey matter (GM) in these conditions 1,3,7–9.

However, performing tissue- and/or column-specific quantitative SC MRI measurements *in vivo* is technically challenging. This is mostly due to the small cross-sectional size of the cord, and the potential for cord motion during the scan, caused both by involuntary patient movement and physiological motion 10,11. As certain neurological conditions have been shown not only to affect cord GM and WM differently, but also to affect the lateral and dorsal columns of the cord preferentially (for example, in motor neuron disease and subacute combined degeneration 12,13), it is desirable to be able to make quantitative measurements from these regions in addition to whole cord measurements.

There is also currently no established method for the co-registration of multi-parametric SC MRI data, although some previous studies have co-registered data of the same contrast acquired either axially 14 or sagittally 15. In addition, some recent studies have performed rigid registration of magnetisation transfer (MT)-weighted data to spoiled gradient echo (fast field echo) images 16 and non-rigid registration of (downsampled) MT-weighted data to *b* = 0 images in a diffusion tensor imaging dataset to enable the quantification of diffusion tensor imaging parameters and magnetisation transfer ratio (MTR) from the same regions of interest (ROIs) 17. As possible cord motion is a significant problem in SC imaging, co-registration is an important issue for quantitative measurement methods and for protocols incorporating different MRI contrasts.

It has been demonstrated previously that myelin content and axonal density in multiple sclerosis cord WM correlate with *T*_1_, *T*_2_, proton density (PD) and MTR values at high field strength 18,19. In addition, previous cervical cord ROI analysis has shown a correlation of tissue-specific values of MTR 14,20 and MTCSF 21 [where the MT-weighted image is normalised by the average cerebrospinal fluid (CSF) signal in the same slice of an MT-weighted scan] with sensory and motor dysfunction. The measurement of such quantitative parameters in the cord *in vivo* could be applicable to many conditions involving the SC. However, MTR is only ‘semi-quantitative’, as it depends on the sequence parameters and is influenced by *T*_1_ relaxation and flip angle inhomogeneities 22. Multi-parameter mapping using multi-echo three-dimensional fast low-angle shot (FLASH) sequences provides a range of MR parameters, including the apparent proton density (APD), *T*_1_, *R*_2_* (=1/*T*_2_*), MTR and a new parameter based on MT [denoted MT saturation and defined in ref. 23], which is robust against *B*_1_ and *T*_1_ effects. The estimation of such a range of quantitative parameters can provide sensitive and specific information on changes in tissue pathology.

In this study, we first aimed to set up a robust co-registration pipeline for intra-subject multi-contrast SC imaging data previously acquired as part of a multi-parameter mapping study 24 using the linear registration tool of the FSL software package (FLIRT; http://www.fmrib.ox.ac.uk/fsl/flirt). This co-registration method was then applied to the three sets of images of different contrast in the cervical cords of each of 13 healthy volunteers. This enabled us to make cervical cord (levels C1–C5) and tissue-/column-specific ROI measurements (in each subject's native space) of parameter values at cord level C2 and to establish the normal values of such quantitative measures *in vivo*. These measurements also allowed us to determine inter-subject variation of the proposed parameters in the SC 25.

## EXPERIMENTAL DETAILS

### MR acquisition

Thirteen subjects (12 men, one woman; aged 36.4 ± 12.3 years) were scanned on a 3-T Magnetom TIM Trio scanner (Siemens Healthcare, Erlangen, Germany) with a head, neck and spine receiver coil combination. For the multi-parameter mapping, three scans were performed on each subject: a slab-selective three-dimensional multi-echo FLASH sequence 23,26 was run three times, with predominantly MT (MTw), PD (PDw) or *T*_1_ (T_1_w) weighting. For each of these three-dimensional scans, eighty 3-mm-thick partitions were acquired, with an axial field of view of 200 mm × 200 mm, acquisition matrix 256 × 256, sinc interpolated in image space to 512 × 512, and phase encoding anterior/posterior (A/P), with parallel imaging acceleration factor 2 in the phase encoding direction. For the PDw (and later PDw and *T*_2_*w) images (TR = 24.05 ms; flip angle *α* = 6°), images were acquired from six gradient echoes at equally spaced TEs between 3.0 and 18.55 ms (with an acquisition bandwidth of 425 Hz/pixel). This value for the echo train length was chosen as it is a trade-off between the provision of purely PDw images, acquiring data at multiple echoes to increase signal-to-noise ratio and simultaneously providing *T*_2_* information. The MTw data (five echoes) were acquired with an additional 4-ms off-resonance Gaussian radiofrequency (RF) pulse (nominal *α* = 220°; offset frequency, 2 kHz) before each excitation pulse, and T_1_w data (five echoes) were acquired with TR = 22 ms and *α* = 20°.

The spatial distribution of the *B*_1_ transmit field was also measured using a modified three-dimensional actual flip angle imaging method 27, with alternative RF/gradient spoiling scheme 28, to enable correction of the *T*_1_ maps. The actual flip angle imaging sequence used excitation pulses with *α* = 60°, alternating TR delays of 50 and 150 ms and a gradient echo readout at TE = 3.05 ms. Forty 6-mm partitions were acquired, with a field of view of 200 mm × 200 mm and acquisition matrix of 64 × 64, i.e. pixel size of 3.13 mm × 3.13 mm, sinc interpolated to 0.39 mm × 0.39 mm, to enable co-registration with the rest of the protocol and subsequent correction of the *T*_1_ maps. The total acquisition time of all the imaging data (including *B*_1_ mapping) was approximately 19 min.

### Intra-subject registration pipeline

Cord levels C1–C5 of each of the healthy subjects were manually identified and those slices were extracted from each of the PDw, T1w and MTw volumes. The multi-echo data were averaged for each contrast to produce an averaged PDw (avePDw), MTw (aveMTw) and T1w (aveT1w) volume. The following registration pipeline was then applied:

1. The avePDw and aveT1w images were registered to the aveMTw data, using FLIRT (http://www.fmrib.ox.ac.uk/fsl/flirt). FLIRT parameters were as follows: degrees of freedom = 6, cost function = normalised mutual information, which is the most suitable cost function for images of different contrast 29, and interpolation = sinc 25.
2. The registered aveT1w image contrast was inverted to match the avePDw/aveMTw images, i.e. such that CSF was brighter than the SC tissue.
3. The registered avePDw and inverted registered aveT1w and aveMTw images were then ‘averaged’ to create a new registration target.
4. Finally, the original avePDw, aveT1w and aveMTw images (input of step 1) were registered to the target (output of step 3) using the same FLIRT registration parameters as in step 1. This four-step process was carried out in order to ensure that minimum bias was introduced into the final maps as a result of different interpolation and smoothing associated with the registration process if only step 1 was performed. We refer to the output images from this final step as regPDw, regT1w and regMTw.

Prior to *B*_1_ map calculation, *B*_1_ data were also co-registered to the regT_1_w data, as output from step 4. These data were chosen as the target for the *B*_1_ registration because of the similar (although not identical) image contrast using the same FLIRT parameters as in step 1. The *B*_1_ maps were then used to enable correction of *T*_1_ maps.

### Image analysis

Processing routines developed for use with SPM8 (http://www.fil.ion.ucl.ac.uk/spm) and FSL (http://www.fmrib.ox.ac.uk/fsl) were employed for analysis 23,26,30,31. *R*_2_* was estimated from the images acquired using the multi-echo ‘PDw’ acquisition sequence (PDw and, for the later echoes, *T*_2_*w images) by linear regression of the logarithm of the signal across the multiple echoes (the transformations of regPDw from step 4 were applied to individual echoes prior to fitting).

For the calculation of the other parameter maps, the output images from step 4 were used, and maps of *T*_1_ and the amplitude APD (not corrected for RF receive inhomogeneities) were calculated from the regPDw and regT_1_w images via a rational approximation of the FLASH signal *S* 30:

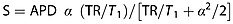
1

In the approximate signal equation of the MT-FLASH experiment:

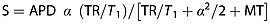
2

The MT saturation parameter was calculated from the regMTw images by inserting the estimated APD and *T*_1_ values in the approximate signal equation for the MT-FLASH experiment 23. The MT saturation parameter represents the additional percentage reduction in the steady-state FLASH signal caused by the saturation effect of a single MT pulse. It is calculated from Equation [2] using APD and *T*_1_ obtained from Equation [1], and has been shown to be insensitive to inhomogeneities in the RF transmit field and receive fields 23. In addition, unlike MTR, the MT saturation parameter is not affected by *T*_1_ relaxation 23.

Furthermore, two subsets of odd and even echoes were analysed following the same processing routines as for the complete data to re-calculate the parameter maps of APD, *T*_1_, MT saturation and MTR, i.e. using only half the data in each case, but with approximately the same effective TE (there is a shift by one echo spacing of odd/even echoes). The resulting maps were compared, and this was assumed to give an assessment of the robustness of parameter value estimation.

### C1–C5 cervical cord analysis

Semi-automatic cord segmentation was performed on the regT_1_w images using a method based on an active surface model 32 implemented in the Jim software library (http://www.xinapse.com). Masks were generated for each subject over the entire cord from levels C1 to C5 and spatial mean parameter values and standard deviations (SDs) were examined to establish inter-subject variation. As for the cervical cord maps, parameter maps for all subjects were re-calculated using subsets of only even or odd echoes acquired for each of the MTw, T_1_w and PDw data.

### Tissue- and column-specific measurement: ROI analysis

Four ROIs were manually outlined by the same single observer (RSS) using the ROI analysis tool in Jim 6.0 in the dorsal, left and right lateral columns and GM over five slices at the C2 level of the cervical cord, similarly to ref. 33, and applied to the parameter maps. First, the five slices centred at the C2 level of the cord were selected using the co-registered sagittally oriented images viewed in Jim. The regMTw images were used for region placement as the highest GM–WM contrast was observed in these images.

### Statistical analysis

IBM SPSS version 20.0 for Windows (SPSS, Inc., Chicago, IL, USA) was used for statistical analysis. Within-subject differences between different ROIs in the GM and WM columns were assessed using paired *t*-tests.

Inter-subject coefficients of variation (CoV) (measured in %) were calculated according to:

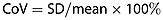
3where mean is the mean parameter value and SD is the standard deviation for all subjects studied.

Intra-subject estimates of the robustness of parameter measurements were calculated similarly using the SD and mean of the two measured values calculated using even or odd echoes, respectively.

## RESULTS

### SC registration pipeline

**Figure 1:**
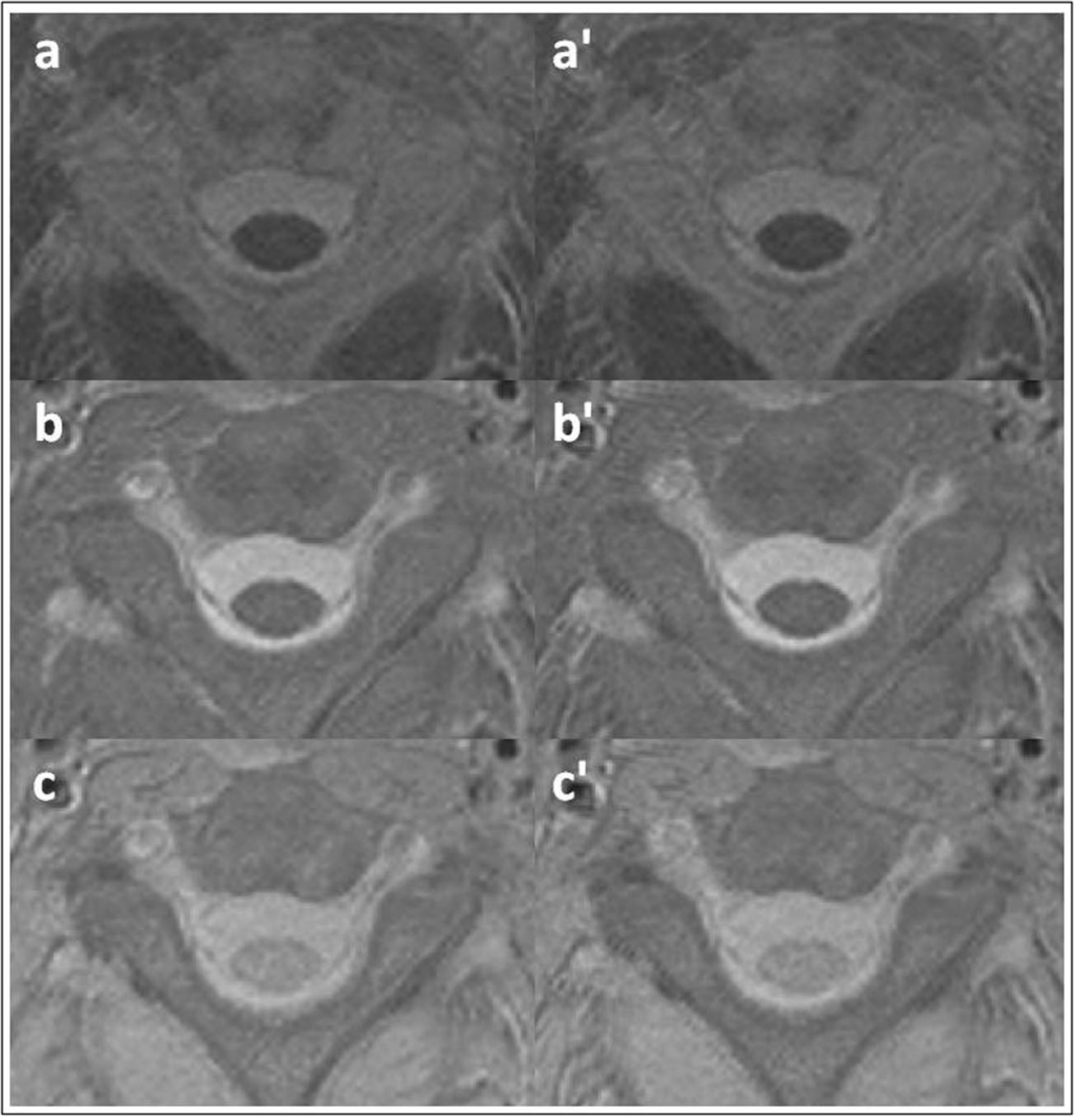
Example normalised subtraction MTw – PDw (a, a′), MTw – T1w (b, b′) and PDw – T1w (c, c′) maps without (left) and with (right) registration.

Example subtraction maps (MTw – PDw, MTw – T1w, PDw – T1w) before (a,b,c) and after (a′,b′,c′) registration (normalised prior to subtraction) are given in Figure 1. Co-registration is essential to make accurate quantitative measurements in the SC, when using a protocol incorporating several scans of more than a few minutes in length 25, and to enable tissue- and column-specific measurements to be made. Co-registration preserves or sometimes enhances the detection of detail in registered images.

### C1–C5 cervical cord analysis

**Figure 2:**
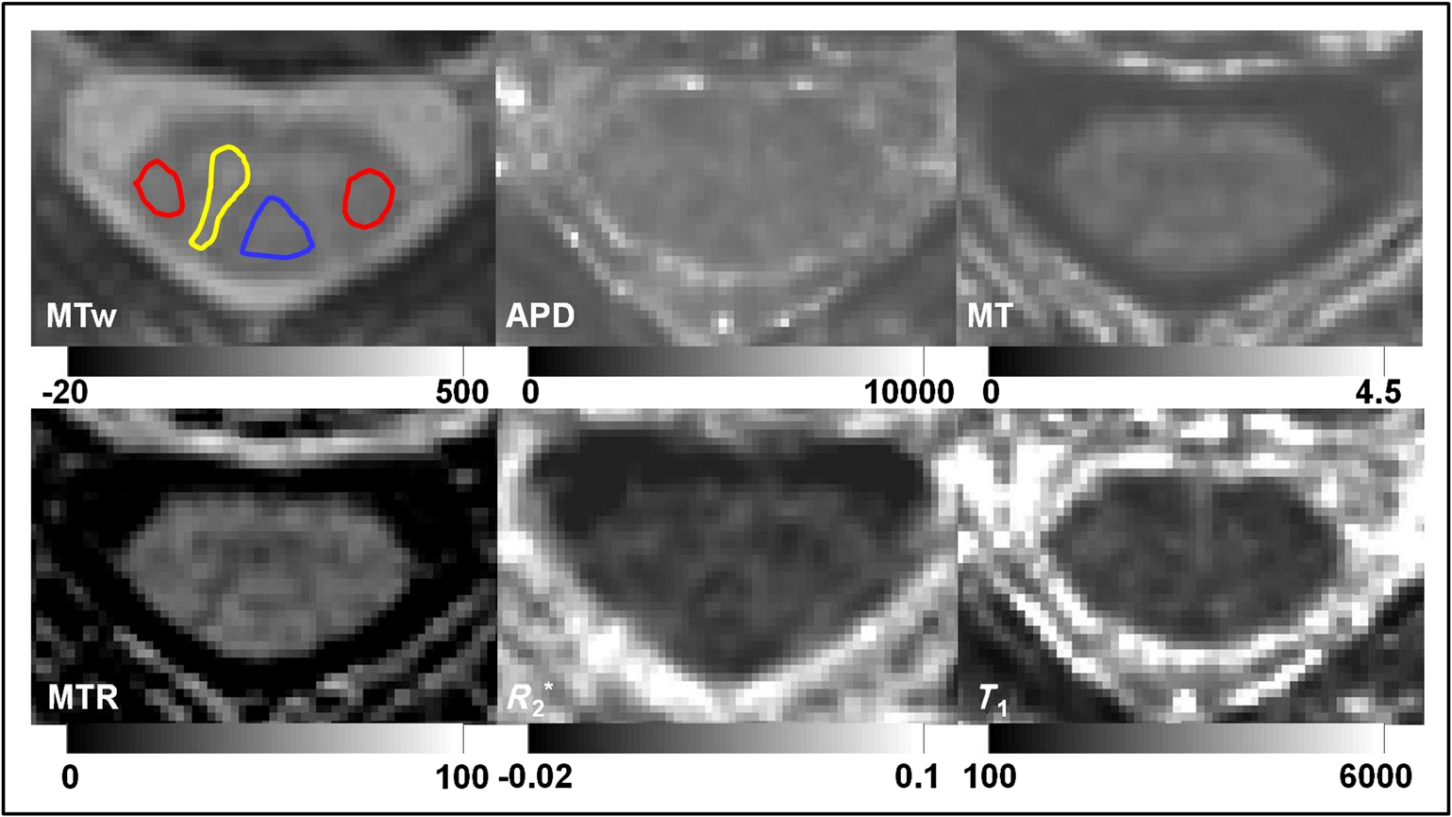
Example magnetization transfer-weighted (MTw) image with approximate region of interest (ROI) placement indicated (red, lateral column; blue, dorsal column; yellow, grey matter) and parameter maps: apparent proton density (APD) (a.u.), MT (p.u.), MT ratio (MTR) (p.u.), *R*_2_* (s^–1^) and *T*_1_ (ms), with grey colour variation bars.

Example APD, *T*_1_, MT saturation, MTR and *R*_2_* maps for a single subject at cervical cord level C2 are shown in Figure 2.

**Figure 3:**
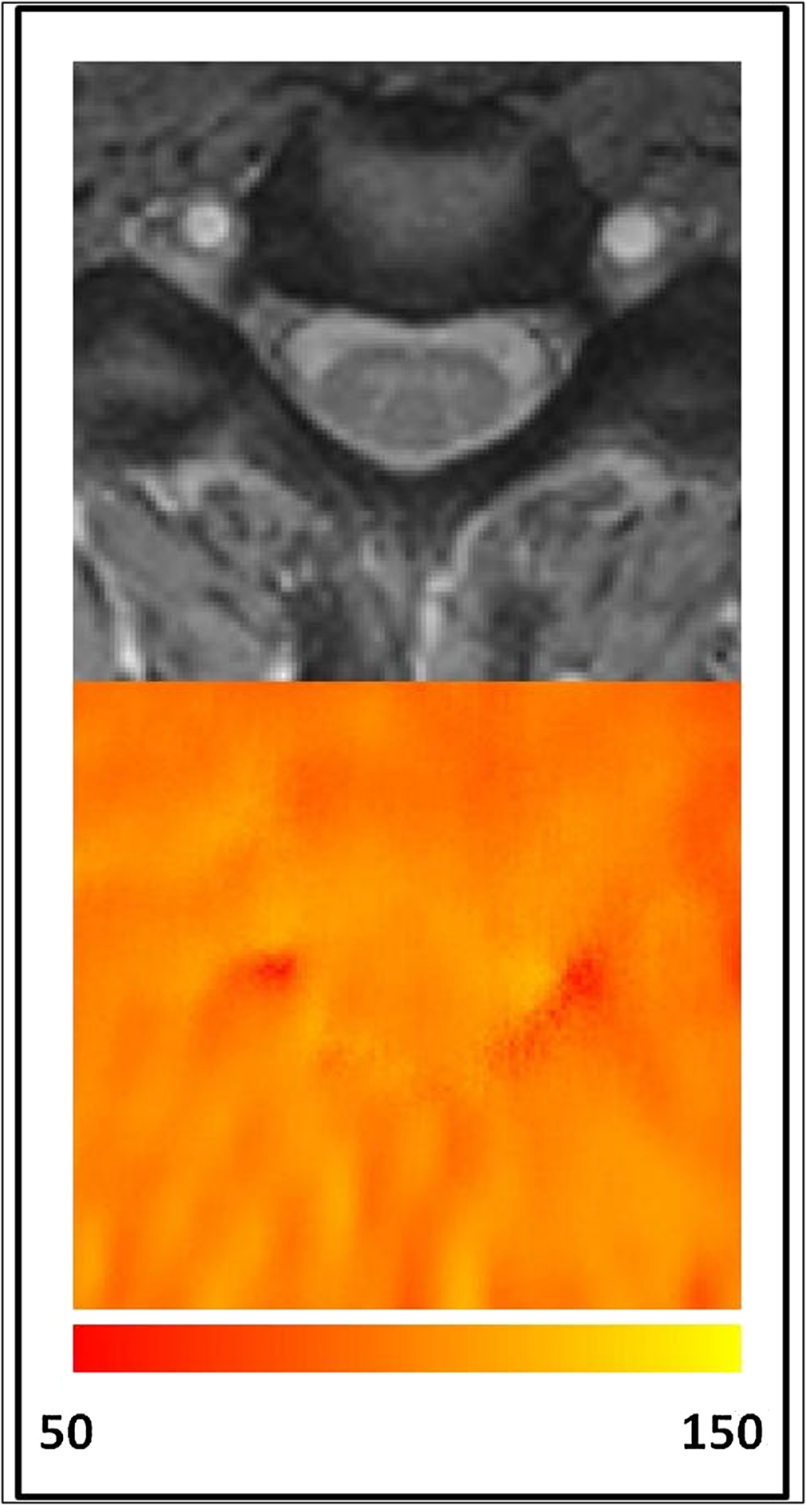
Example magnetization transfer-weighted (MTw) image from the same single subject, with matching *B*_1_ map [and colour bar indicating *B*_1_ value in per cent (of nominal value)].

In Figure 3, a *B*_1_ map for the same single subject is shown, again at cord level C2 (with the MTw image to indicate positioning).

GM and WM appear to be better differentiated in the MT saturation maps than in the MTR maps, which could potentially indicate enhanced sensitivity to macromolecular content differences in tissue, in line with a reduced sensitivity to *B*_1_ and *T*_1_ in the MT saturation maps in comparison with the MTR maps 22–24.Post-mortem validation with histology is required to confirm the biophysical basis of the MT saturation parameter map contrast in comparison with MTR. In addition, other factors, such as the particular sequence acquisition parameters, could have contributed to the contrast observed in parameter maps.

Table 1 gives the cord parameter mean values (with SDs in parentheses) measured over cord levels C1–C5. Mean cervical cord MTR values are consistent with previously reported 3-T measurements 34, and other parameters are similar to those obtained using the same technique in the brain at 3 T 22,26,35 (the acquisition parameters were not identical to those used in this protocol, and therefore the MT effect would be slightly different from this study).

**Table 1:** Mean cervical cord (levels C1–C5) parameter values for 13 healthy volunteers

Parameter	Mean parameter value (±SD)	Cross-subject median robustness of parameter estimation (range) (%)
APD (a.u.)	4822 (±718)	6.14 (2.22–7.23)
*T*_1_ (ms)	1848 (±143)	7.08 (3.30–16.2)
MT saturation (p.u.)	1.40 (±0.12)	7.74 (4.37–11.4)
MTR (p.u.)	40.4 (±1.53)	3.95 (0.26–7.29)
*R*_2_^*^ (s^–1^)	22.6 (±1.53)	6.23 (1.10–10.5)

APD, apparent proton density; MT, magnetization transfer; MTR, magnetization transfer ratio; SD, standard deviation.

C1–C5 level cervical cord CoVs for MTR and MT saturation measurements are 6.8% and 8.3%, respectively. The *R*_2_* and *T*_1_ CoVs are also low (6.8% and 7.7%, respectively), but the CoV in APD is larger than that of all other parameters (14.9%).

Estimates of the robustness of parameter measurements, as obtained by repeating the processing with subsets of odd or even echo data, are given in Table 1. These estimates of the robustness of parameter estimation are expected to be somewhat low, probably as they were only based on half the data in each case. However, these data represent the ‘intra-scan’ reproducibility, which cannot be compared directly with the inter-scan reproducibility. Intra-class correlation coefficients for each parameter were as follows: APD, 0.91; MT saturation, 0.58; MTR, 0.40; *R*_2_*, 0.76; *T*_1_, 0.76.

### ROI analysis

Example MTw images (with ROI placement indicated) and parameter maps from a single subject at cervical cord level C2 are shown in Figure 2. Mean tissue-specific parameter values are given in Table 2. All parameter values were shown to be significantly different in GM relative to WM regions via paired *t*-tests (*p* < 0.05). No significant differences were observed in any parameters between left and right lateral WM, but *R*_2_* was found to be significantly different in dorsal WM compared with both right and left lateral WM (*p* < 0.05), and *T*_1_ was significantly lower in left lateral WM than in dorsal WM.

**Table 2:** Mean tissue- and column-specific parameter values for 13 subjects

Region of interest	APD (±SD) (a.u.)	MT saturation (±SD) (p.u.)	MTR (±SD) (p.u.)	*R*_2_^*^ (±SD) (s^–1^)	*T*_1_ (±SD) (ms)
Dorsal WM	4668 (±699)	1.43 (±0.15)	44.5 (±1.9)	22.3 (±3.04)	1735 (±205)
Left lateral WM	4408 (±583)	1.47 (±0.15)	44.6 (±2.2)	21.2 (±2.61)	1593 (±221)
Right lateral WM	4792 (±947)	1.43 (±0.15)	43.7 (±2.5)	20.5 (±2.35)	1707 (±219)
GM	5160 (±910)	1.18 (±0.11)	40.6 (±2.6)	18.9 (±1.96)	1815 (±170)

APD, apparent proton density; GM, grey matter; MT, magnetization transfer; MTR, magnetization transfer ratio; SD, standard deviation; WM, white matter.

Maximum inter-subject CoVs for region-specific measurements were 19.8% for APD, 13.7% for MT saturation, 5.8% for MTR, 13.6% for *R*_2_* and13.9% for *T*_1_ in WM, and 17.6% for APD, 9.8% for MT saturation, 6.4% for MTR, 10.4% for *R*_2_* and 9.4% for T_1_ in GM.

## DISCUSSION

We investigated the possibility of performing quantitative multi-parametric measurements in the GM and WM columns of the healthy SC at 3 T. In order to perform region-specific measurements, it was necessary to incorporate an intra-subject co-registration pipeline for the multi-modal cervical cord data prior to the estimation of multi-parametric maps in 13 healthy subjects. Parameter values in different WM columns and GM regions were measured over five slices at the C2 cervical level, in addition to cervical cord measurements over levels C1–C5. Significant differences in the parameter values between WM and GM regions were found, demonstrating that it is feasible to make tissue-specific multi-parametric measurements in the cord using the proposed post-processing pipeline. Future work will include an investigation of the registration method in pathology, as well as the investigation of SC registration for other MRI contrasts.

The method described here provides measurements of several quantitative and semi-quantitative MRI parameters at 3 T in the cervical cord in a clinically feasible time (<20 min in total). WM parameter values are comparable with those obtained previously for the whole SC without registration of the cord 24, and WM MTR values are similar to those obtained previously at 3 T in the brain, with a similar protocol but slightly different acquisition parameters 23,26, and cord (using alternative methods) 34, demonstrating the accuracy of the technique.

MTR values measured in this study have a mean of 44.5 ± 1.9 p.u. in cervical cord dorsal WM, which is lower than the values measured in a recent upper cervical cord (C2–C3 levels) MTR study of 10 healthy volunteers 16 (mean value of 51.4 ± 1.5 p.u. in cord WM), and higher than those measured over the cervical cord levels C4–C7 in 21 healthy volunteers by Cohen-Adad *et al*. 36 (mean values of approximately 32 p.u. in the lateral columns and 33 p.u. in the dorsal columns). However, the MTR is a ‘semi-quantitative’ measure which reflects a complex combination of various biological factors. It is also highly dependent on the RF transmit field, sequence parameters and the MR system, and is therefore difficult to compare across centres 37.

The MTR was found to be significantly different in GM relative to lateral and dorsal WM columns in the quantitative MT study performed by Smith *et al*. 33 at 1.5 T, and this work confirms that finding. Yiannakas *et al*. 16 measured cord WM and GM MTR at cervical level C2 at 3 T, and also found the WM MTR to be significantly higher than the GM MTR. Lateral and dorsal column MTR values were also compared, but these were not found to be statistically significantly different, in agreement with our study. Similarly, in another 3-T study, performed by Smith *et al*. 38 in 2010, dorsal and lateral column WM MTR values were not found to be significantly different. Although the axonal density is lower in the lateral columns, the myelin content may be similar in the lateral and dorsal columns, and the MTR may reflect myelin content more than axonal density 39.

It should also be noted that this protocol was not designed to map small *R*_2_* value differences between GM and WM, as the longest TE is less than 20 ms. In addition, *R*_2_* was estimated from the logarithm of the signal intensities in the PDw images (acquired at different TEs) using a linear regression. As the signal-to-noise ratio of the different echoes varies, heteroscedasticity may have had an impact on the fit. The assumption of monoexponential signal decay may be violated in some areas [e.g. those suffering from susceptibility artefacts 40]. This could have introduced some bias in both the APD and *R*_2_* maps in such regions, where differences in shim and head positioning between subjects may have affected the results.

*T*_1_ values measured here are longer than those measured previously in the cord at 3 T 34,41 and in brain 42,43 via different methods. However, other studies have also measured higher *T*_1_ values in the brain 44,45, close to those in our study. Our *T*_1_ values were also slightly closer to those measured in the brain using the same multi-parameter mapping method 35 (0.96 s and 1.64 s in WM and GM, respectively). There are a number of possible reasons for this disparity.

First, the *B*_1_ maps measured in this study were rather flat, with *B*_1_ values close to 100% of the nominal value, suggesting that the *B*_1_ mapping method used is not very sensitive in the SC (see Fig. 3).

In addition, flow effects and *B*_0_ inhomogeneities were not accounted for in this study, which could have contributed to errors in the measured parameter values. It is also possible that the spatial characteristics of water diffusion in the cord may have influenced the FLASH spoiling behaviour, affecting the measured quantitative parameter values 46.

However, the good inter-subject reproducibility of the *T*_1_ values (7.7% for cervical cord measurements at levels C1–C5) means that they could be used to detect changes caused by pathology in the cervical cord.

Inter-subject CoVs are low and comparable with literature values. C1–C5 level cervical cord CoVs for MTR and MT saturation are 6.8% and 8.3%, respectively, which are lower than those observed in a reproducibility study of another MT measure (MTCSF) applied in the SC at 3 T (approximately 10%) 38. The *R*_2_* and *T*_1_ CoVs are also low (6.8% and 7.7%, respectively), but the CoV of APD is larger than that of all other parameters (14.9%).

Maximum inter-subject CoVs for tissue-specific measurements were 19.8% for APD, 13.7% for MT saturation, 5.8% for MTR, 13.6% for *R*_2_* and13.9% for *T*_1_ in WM, and 17.6% for APD, 9.8% for MT saturation, 6.4% for MTR, 10.4% for *R*_2_* and 9.4% for *T*_1_ in GM. A recent study that measured *T*_1_ and *T*_2_ in WM and GM regions in the cervical cord at 3.0 T found both the *T*_1_ and *T*_2_ reproducibility to be between 5% and 10%, but these values were for a single slice at the C3 level in six volunteers, and so are not directly comparable with the results presented here 41, and *T*_2_ and *T*_2_* values are very different in the SC. In addition, in our study, five parameters were estimated with data acquired in a total scan time of only 19 min, compared with about 27 min to measure *T*_1_ and *T*_2_ alone in the study by Smith *et al*. 35. As data were only acquired from a single slice in that particular study, there are several factors that could have contributed to the differences observed in reproducibility between that study and our work. These include the different numbers of shots and phase sensitivity, flow effects, resolution (affecting the *B*_0_ sensitivity) and possible slice profile effects, in addition to the differing acquisition times of the sequences.

The low intra-subject variation in the mean parameter values (0.26–16.2% for all parameters in all subjects, but much less than 10% in 10 of 13 subjects in all parameters) also demonstrates the intra-scan reproducibility of the measurements made using this method. In the study performed by Smith *et al*. 38, intra-subject variation was examined by acquiring MTCSF data from nine healthy volunteers on two separate occasions separated by at least 7 days. Mean values were calculated over the whole columns (22 slices; with right and left lateral column values averaged), and the Bland–Altman difference 47 was calculated for the two repeated scans, and ranged from 0.85% to 8.44% for the lateral column and 0.05% to 5.32% for the dorsal column.

The intra-class correlation coefficients for MTR and MT saturation are rather low. This is likely to be a result of the low variability observed between subjects, as evidenced by the low inter-subject CoVs (6.8% and 8.3% for MTR and MT saturation, respectively). Further studies should include measurements in patient populations in which the inter-subject variation would be expected to be larger.

It should be noted, however, that these values cannot necessarily be thought of as precise measures of the intra-class correlation coefficient as only half the data were used for each of the two measures (odd/even echoes), rather than repeating the whole measurement twice. It should also be noted that the MT saturation measures are based on three acquisitions without additional degrees of freedom in the estimation. Thus, they are usually noisier than the *T*_1_ or APD estimates 35. It is also likely that heteroscedasticity (discussed above) affected the APD and *R*_2_* estimates.

As ROIs placed in WM and GM columns in the SC are very small, displacements of just one to two voxels would render quantitative tissue-specific measurements in the cord impossible, and cord motion of this order could easily be expected given the duration of the scans acquired here. Therefore, these measurements would not have been possible without the use of co-registration. The use of immobilisation methods, such as MR-compatible collars, could provide further stability of the subject throughout the scan, and possibly improve alignment between subsequent scans within a single scanning session 16.

Transmit *B*_1_ inhomogeneity was accounted for by *B*_1_ correction in the maps of APD and *T*_1_, although, as discussed above, *B*_1_ maps were flattened, which we believe may have contributed somewhat to the high *T*_1_ values observed in this study_._ However, receive inhomogeneity is not accounted for in the APD maps and would have contributed to the inter-subject variation observed, as a result of different positioning of the subjects. The inclusion of receive sensitivity mapping in the protocol may be desirable, but conventional approaches based on the assumption of reciprocity are problematic at higher fields, such as 3 T 41. Slab-selective excitation was used here to avoid signal wrap-around, in contrast with the non-selective excitation of the same technique applied in the brain 22,30. Although inhomogeneities in the transmit/receive RF field are intrinsically compensated in the MT saturation parameter, we could not assume that transmit *B*_1_ errors are optimally cancelled in all areas. In addition, it is possible that slab profile effects may have affected the *B*_1_ correction of the *T*_1_ maps.

The technique described is rapid and provides several quantitative MRI measures, which could be applied to examine changes in studies of neurological diseases affecting the SC, either on a region of interest or volumetric basis. In addition to standard relaxation parameters, the MT saturation parameter is of particular interest as, unlike the MTR 22, it is minimally affected by *T*_1_ relaxation, is less sensitive to *B*_1_ inhomogeneities, and GM–WM contrast in MT saturation maps is increased relative to that in MTR maps. MT saturation maps have been used previously for the segmentation of GM structures in the brain 31, in studies of the cortical spinal tract after SCI 48 and in healthy ageing 49, and may also prove to be useful in this context in the SC. This quantitative multi-parametric measurement method has the potential to provide insight into the pathological processes occurring in the cervical cord affected by various neurological disorders, such as multiple sclerosis, amyotrophic lateral sclerosis, neuromyelitis optica and SCI.

## Abbreviations

A/P: anterior/posterior
APD: apparent proton density
aveMTw: average MT-weighted
avePDw: average PD-weighted
aveT1w: average T_1_-weighted
CoV: coefficient of variation
CSF: cerebrospinal fluid
FLASH: fast low-angle shot
GM: grey matter
MT: magnetisation transfer
MTCSF: MT-weighted image normalised by the average CSF MT-weighted signal
MTR: magnetisation transfer ratio
MTw: MT-weighted/weighting
regMTw: registered MT-weighted
regPDw: registered PD-weighted
regT1w: registered T_1_-weighted
RF: radiofrequency
PD: proton density
PDw: PD-weighted/weighting
ROI: region of interest
SC: spinal cord
SCI: spinal cord injury
SD: standard deviation
T_1_w: T_1_-weighted/weighting
WM: white matter
